# Impaired Platelet Aggregation and Rebalanced Hemostasis in Patients with Chronic Hepatitis C Virus Infection

**DOI:** 10.3390/ijms18051016

**Published:** 2017-05-08

**Authors:** Nick S. Nielsen, Sofie Jespersen, Julie C. Gaardbo, Caroline J. Arnbjerg, Mette R. Clausen, Mette Kjær, Jan Gerstoft, Vibe Ballegaard, Sisse R. Ostrowski, Susanne D. Nielsen

**Affiliations:** 1 Department of Infectious Diseases, University Hospital of Copenhagen, Rigshospitalet, 2100 København, Denmark; nick@nielsen.mail.dk (N.S.N.); sofie.dikeledi@gmail.com (S.J.); juliegaardbo@hotmail.com (J.C.G.); caroline.arnbjerg@gmail.com (C.J.A.); Jan.Gerstoft@regionh.dk (J.G.); vibececilie@gmail.com (V.B.); 2 Department of Hepatology, University Hospital of Copenhagen, Rigshospitalet, 2100 København, Denmark; anne.mette.rye.clausen@regionh.dk (M.R.C.); mette.skalshoei.kjaer@regionh.dk (M.K.); 3 Department of Clinical Immunology, University Hospital of Copenhagen, Rigshospitalet, 2100 København, Denmark; sisse.ostrowski@gmail.com

**Keywords:** HCV, hemostasis, platelet aggregation

## Abstract

Increased risk of both cardiovascular disease (CVD) and bleeding has been found in patients with chronic hepatitis C (CHC) infection, and a re-balanced hemostasis has been proposed. The aim of this study was to investigate functional whole blood coagulation and platelet function in CHC infection. The prospective study included 82 patients with CHC infection (39 with advanced liver fibrosis and 43 with no or mild liver fibrosis) and 39 healthy controls. A total of 33 patients were treated for CHC infection and achieved sustained virological response (SVR). Baseline and post-treatment blood samples were collected. Hemostasis was assessed by both standard coagulation tests and functional whole blood hemostatic assays (thromboelastograhy (TEG), and platelet aggregation (Multiplate). Patients with CHC and advanced fibrosis had impaired platelet aggregation both compared to patients with no or mild fibrosis and to healthy controls. Patients with CHC and advanced fibrosis also had lower antithrombin, platelet count, and coagulation factors II-VII-X compared to healthy controls. In contrast, TEG did not differ between groups. In treated patients achieving SVR, post-treatment platelet count was higher than pre-treatment counts (*p* = 0.033) and ADPtest, ASPItest, and RISTOhightest all increased post treatment (all *p* < 0.05). All Multiplate tests values, however, remained below those in the healthy controls. CHC-infected patients displayed evidence of rebalanced hemostasis with only partly hemostatic normalization in patients achieving SVR. The implications of rebalanced hemostasis and especially the impact on risk of CVD and bleeding warrants further studies.

## 1. Introduction

Hepatitis C virus (HCV) is the cause of viral hepatitis, with an estimated 130–150 million persons with chronic hepatitis C infection (CHC) worldwide [1]. Approximately 80% of those infected will develop CHC, and 10–15% of patients with CHC infection will develop advanced liver disease with cirrhosis and increased risk of hepatocellular carcinoma (HCC) [2]. Besides these well-known manifestations of CHC, increased risk of cardiovascular disease (CVD) and thromboembolic events has been documented [3,4,5,6]. Vascular inflammation and altered coagulation caused by CHC infection has been hypothesized to contribute to increased CVD risk. In contrast, CHC infection is also associated with thrombocytopenia and lower levels of coagulation factors, contributing to an increased risk of bleeding [7,8,9]. Evidence thus points towards manifestations of both hypo- and hypercoagulability.

Hemostasis is balanced by pro- and anticoagulant and pro- and antifibrinolytic factors, most of these being synthesized by the liver [10]. Advanced liver disease is thus associated with perturbations in the level of these due to secretory deficiencies [11,12]. Furthermore, lower platelet count, lower concentrations of factor II-VII-X, and anti-fibrinolytic factors are all features of CHC infection, suggesting hypocoagulability [7,10,13]. However, higher concentrations of von-Willebrand factor (vWf) and Factor VIII as well as lower concentrations of anticoagulant factors including Protein C and S have also been reported in CHC infection suggesting hypercoagulability [10,13,14,15]. The combination of these alterations may lead to a rebalanced hemostasis as a common explanation for the observed increased risk of both thromboembolic events and bleeding in patients with CHC infection [13]. 

Conventional plasma-based coagulation tests such as activated partial thromboplastin time (APTT) and international normalized ratio (INR) are often used to assess both hemostasis and liver function in CHC-infected patients [16]. However, these tests are poor predictors of bleeding risk and cannot predict thrombotic events [16]. In contrast, the risk of bleeding and thromboembolic events can be assessed by functional hemostatic whole blood tests such as thromboelastography (TEG) and impedance platelet aggregometry (Multiplate), which are well-established tests reflecting secondary and primary hemostasis, respectively [13,15,17]. To the present authors’ knowledge, no previous studies have investigated whole blood hemostatic function in patients with CHC infection.

The primary aim of this study was to investigate hemostasis in patients with CHC infection using functional hemostatic whole blood tests and determine possible associations with liver fibrosis. CHC-infected patients with no or mild and with advanced fibrosis as well as a group of uninfected controls were included in a cross sectional study. To determine possible effects of HCV viral replication on coagulation, a prospective study of patients starting treatment against CHC infection was conducted. It was hypothesized that untreated CHC infection was associated with altered whole blood functional hemostasis tests and that hemostasis would normalize after successful treatment of HCV. 

## 2. Results

The three groups (CHC with no or mild fibrosis, CHC with advanced fibrosis, and healthy controls) in the cross sectional study differed regarding age (Table 1). A Spearman correlation test did not reveal any significant correlations between age and TEG or Multiplate data. Furthermore, due to study design, the CHC-infected group with advanced fibrosis had a higher level of fibrosis compared to patients with no or mild fibrosis (Table 1).

### 2.1. Altered Standard Coagulation Tests in CHC-Infected Patients

Compared to healthy controls, patients with CHC infection, both with no or mild fibrosis and with advanced fibrosis, had lower platelet counts and lower concentration of antithrombin, whereas only patients with advanced fibrosis had lower concentration of coagulation factor II-VII-X (Table 2). 

Compared to CHC-infected patients with no or mild fibrosis, CHC-infected patients with advanced fibrosis had lower platelet counts (median 139 × 10^9^/L versus 232 platelets × 10^9^/L, *p* < 0.001) and lower concentration of coagulation factors (0.76 arb.units versus 0.88 arb.units, *p* = 0.002). Furthermore, the concentration of antithrombin was lower in patients with advanced fibrosis compared to patients with only no or mild fibrosis (0.86 IU/L versus 1.01 IU/L (*p* < 0.001)). The proportion of patients with D-dimer above threshold was 21% in the group of CHC-infected patients with advanced fibrosis versus 2% in CHC-infected patients with no or mild fibrosis (*p* = 0.009). APTT and fibrinogen did not differ between CHC-infected patients (Table 2). A sub-group analyses of patients with cirrhosis (Fibroscan >12 kPa or F4 verified with biopsy) vs. no or mild fibrosis is shown in Table S1, and results are comparable to advanced fibrosis versus no or mild fibrosis presented in Table 2.

### 2.2. Normal Functional Whole Blood Hemostasis in CHC-Infected Patients

Whole blood hemostasis assed by TEG did not differ between patients with CHC infection and healthy controls (Table 2). However, TEG MA values were lower in CHC-infected patients with advanced fibrosis compared to CHC-infected patients with no or mild fibrosis (58 versus 61 mm, *p* = 0.04). R time, Angle, and Ly30 did not differ between any of the groups (Table 2).

### 2.3. Impaired Whole Blood Platelet Aggregation in Patients with CHC Infection

**Figure 1:**
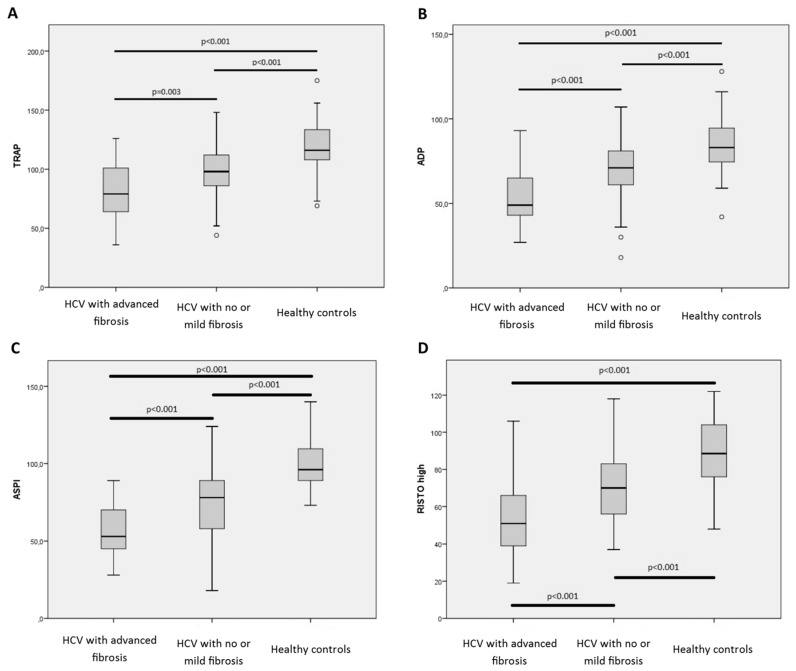
Results from Multiplate analysis preformed on included patient (HCV with no or mild fibrosis *n* = 43 and HCV with advanced fibrosis *n* = 39) and healthy controls (*n* = 39). Boxplot indicating upper and lower quartile (IQR) and median. Whiskers indicating upper and lower extreme. (**A**): TRAP analysis; (**B**): ADP analysis; (**C**): ASPI analysis; (**D**): RISTO high analysis. *p*-values indicate comparison of the groups using Mann-Whitney *U* test.

Patients with CHC infection and advanced fibrosis had impaired platelet aggregation assessed by Multiplate when compared to both CHC-infected patients with no or mild fibrosis and healthy controls (Table 2). TRAP results for CHC-infected patients with advanced fibrosis was 79 units versus 98 units in patients with no or mild fibrosis (*p* = 0.003) and 116 units in the healthy controls (*p* < 0.001). Same pattern was observed for ADP, ASPI and RISTOhigh analysis (Figure 1).

When correcting for platelet count (“multiplate variable”/”platelet count in patient” = multiplate unit per platelet), patients with advanced fibrosis had higher per platelet function than both CHC-infected patients with no or mild fibrosis (TRAP/platelet count = 0.51 versus 0.42 U/10^9^/L *p* = 0.008) and healthy controls (TRAP/platelet count = 0.51 versus 0.46 U/10^9^/L *p* = 0.044). Same pattern was observed for ADPtest but not ASPItest and RISTOhigh test (data not shown).

### 2.4. Minor Changes in Functional Hemostasis after Treatment

A total of 33 patients were treated for CHC infection and achieved SVR. End of treatment samples were compared with baseline samples. Standard blood coagulation tests, platelet counts, and fibrinogen were partly but not fully restored and remained below the healthy controls (Table 3). No changes in overall wholeblood coagulation (TEG analysis) were found (Table 3).

**Figure 2:**
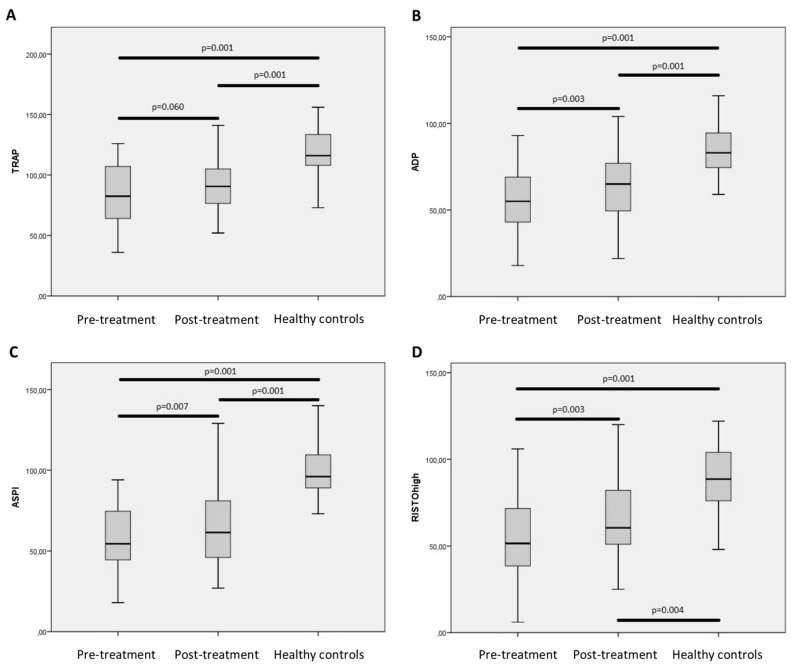
Results from Multiplate analysis preformed on included patient who received treatment against chronic hepatitis C infection and who achieved sustained virological response (*n* = 33). Compared with Multiplate analysis preformed on healthy controls (*n* = 39). Boxplot indicating upper and lower quartile (IQR) and median. Whiskers indicating upper and lower extreme. (**A**): TRAP analysis; (**B**): ADP analysis; (**C**): ASPI analysis; (**D**): RISTO high analysis. *p*-values indicate comparison of the groups using Wilcoxon test or Mann-Whitney *U* test as appropriate.

Improvements were observed in the Multiplate analysis with partly normalization of (*p* = 0.003), ASPItest (*p* = 0.007), and RISTOhigh test (*p* = 0.003). No significant differences were observed in the TRAPtest (*p* = 0.060) (Figure 2). When correcting for platelet count, the per platelet function did not significantly change post treatment when compared to pretreatment (TRAP/platelet count = 0.51 versus 0.49 U/10^9^/L *p* = 0.537). The same pattern was observed for ADPtest, ASPItest, and RISTOhigh test (data not shown).

Compared to uninfected controls, patients who had been treated for CHC infection and achieved SVR still had lower platelet count (*p* = 0.001) and coagulation factor II-VII-X (*p* = 0.001). In contrast whole blood hemostasis was comparable to uninfected control (Table 3). Importantly, patients with SVR still had impaired platelet aggregation with lower TRAPtest (*p* = 0.001) ADPtest (*p* = 0.001), ASPItest (*p* = 0.001), and RISTOhigh test (*p* = 0.004) when compared to healthy controls (Figure 2). Patients who achieved SVR continued to have significant lower per platelet activation in TRAP analysis post treatment when compared to healthy controls (TRAP/platelet = 0.49 versus 0.46 U/10^9^/L *p* = 0.027). Same pattern was observed for ADPtest (*p* = 0.001) and RISTOhigh test (*p* = 0.036) but not ASPItest (*p* = 0.270).

## 3. Discussion

Patients with CHC infection and advanced fibrosis had decreased platelet count and impaired platelet aggregation when compared to both CHC patients with no or mild fibrosis and healthy controls. However, patients with CHC infection and advanced fibrosis also displayed evidence of no overall impairment in functional hemostasis indicating a rebalanced overall hemostatic capacity Importantly, CHC-infected patients that were treated and achieved SVR only obtained partial normalization of both platelets count and platelet aggregation, when compared to the healthy controls.

Coagulation in patients with CHC infection has often been evaluated using standard coagulation tests including factor II-VII-X, INR, and APTT. However, the use of these tests in patients with liver disease has been questioned as the tests are poor predictors of both bleeding and cardiovascular comorbidity [5,16]. The accumulating evidence of increased risk of thromboembolic events and CVD in patients with CHC infection warrants application of tests that measure functional hemostatis [16,18]. TEG has been used for a decade to monitor and goal-direct hemostatic therapy in liver transplantation, cardiac surgery, intensive care, and bleeding in trauma patients [19,20,21,22,23]. TEG has also been used to predict cardiovascular events like pulmonary embolism, myocardial infarction, and deep vein thrombosis (DVT) in post-surgical patients [19,21,24,25] and to evaluate risk of bleeding in patients with liver disease [16,26]. Studies have found an overall normal TEG in patients with liver disease, further suggesting a rebalanced hemostasis [27]. Multiplate has been used in clinical routine to monitor the effect of anti-platelet drugs in surgical settings and platelet dysfunction in critical illness including trauma and sepsis [17,28,29] as well as to evaluate risk of cardiovascular events and effect of antithombotic medication [30,31,32]. Thus, whole blood functional hemostasis tests may provide additional information about hemostasis and CVD risk in CHC-infected patients. At the University Hospital of Copenhagen, TEG and Multiplate are used to goal-direct transfusion and therapy with pro-hemostatic drugs in bleeding or at risk of bleeding patients and in patients prior to high risk surgical procedures [33]. The present study does not assess the possible benefits of using TEG and/or Multiplate in CHC patients in a surgical setting; further studies are needed to assess this aspect. Using whole blood coagulation test, especially Multiplate, could prove valuable in the clinical assessment of liver disease, especially when complicated with bleeding, but further studies are needed aiming to investigate this CHC patients.

Assessing fibrosis using fibroscan to is feasible, but exact cut-off for fibrosis and cirrhosis are still a matter of debate. According to the European Association for the Study of the Liver (EASL) guidelines, values between 5.2 and 9.5 kPa have been proposed as cut-off for ≥F2 fibrosis and values between 11.9 and 14.8 kPa as cut-off for cirrhosis [34]. Based on these guidelines and guidelines from our clinic, the present study divided patients with CHC infection in two groups. One with Fibroscan <8 kPa termed “CHC patients with no or mild fibrosis level” and one with Fibroscan >8 kPa termed “CHC patients with advanced fibrosis” including cirrhosis.

The prevalence of thrombocytopenia in patients with chronic liver disease, including CHC, has been reported to be between 15–70%, with thrombocytopenia being associated with the severity of disease [7,35]. In agreement with this, the present study found lower platelet counts in patients with advanced fibrosis as compared to patients with no or mild fibrosis. Furthermore, patients with advanced fibrosis had impaired platelet aggregation compared to patients with no or mild fibrosis and healthy controls. Likewise, previous studies of platelet function in CHC, applying the platelet function analyzer (PFA-100) and quantitative measurements of metabolites from the platelet metabolism, have suggested a dysfunction of platelets in patients with advanced liver disease [36,37,38]. Thus, impaired function of platelets in patients with cirrhosis due to impaired inositol lipid and arachidonic acid metabolism has been reported [38]. Furthermore, lower platelet activation in patients with hepatitis B and C was found after stimulated with TRAP but not ADP [39]. Interestingly, the present study found higher per-platelet activation in patients with advanced fibrosis compared to that in healthy controls suggesting a compensatory mechanism in patients with advanced liver disease. The present study, however, does not provide information on the exact mechanism for such a compensation, and further studies are needed to explore this subject.

Previous studies have found impairment of both the pro- and anticoagulant system in patients with chronic liver disease, including CHC infection, and a hypothesis suggesting a rebalanced coagulation has been proposed [9,13,14,15]. The present study provides support for this hypothesis. Thus, despite having lower platelet count, impaired overall platelet aggregation, and lower concentration of pro-coagulant factors, patients with CHC infection had normal whole blood functional hemostasis when measured with TEG. This suggests that CHC-infected patients have a rebalanced hemostatic system possible partly due to lower levels of antithombin and by higher per-platelet activity found in present study.

The effect of HCV viral replication on coagulation was assessed in a prospective study of 33 patients that were treated for CHC infection and achieved SVR. Only minor changes were observed in standard coagulation parameters post treatment. Thus, although platelet counts and level of coagulation factor II-VII-X increased post treatment, they remained lower compared to healthy controls. A previous study including 100 patients with SVR showed a slow rise and only gradual normalization in platelet count over 7.5 years of follow up. [40]. Likewise, improvement of platelet aggregation was found in post treatment though it did not reach a level comparable with that in healthy controls. This calls into question whether altered coagulation in CHC infection is merely a direct result of HCV viral replication. An alternative explanation could be damage to the liver as evidenced by the presence of persistent liver fibrosis in patients with SVR. Earlier studies have suggested a slow regression of fibrosis in some but not all patients who achieve SVR [41,42,43,44,45]. Unfortunately, this study was not able to measure the level of fibrosis post treatment. However, the above suggests a long recovery phase beyond that of achieving SVR, indicating the need to evaluate the functional hemostatic system in studies with longer follow-up.

The present study had some limitations. First, it included a relatively low number of participants especially in the prospective study. The cross-sectional design used in part of the study limits analysis of causality. Treated patients only had one blood sample drawn post treatment, limiting the possible effects of long-term SVR on the coagulation system and unfortunately patients with or without fibrosis differed with regards to age. However, significant correlations between age and the functional hemostasis tests were not found. Finally, all hemostasis tests were performed on blood in vitro, not taking the contribution of the endothelium into account.

## 4. Methods

### 4.1. Patients

A cross-sectional study was conducted including 82 CHC-infected patients (*n* = 39 with advanced fibrosis and *n* = 43 with no or mild fibrosis) and 39 uninfected controls. The patients were recruited from The Department of Infectious Diseases and The Department of Hepatology, Copenhagen University Hospital, Rigshospitalet during the period September 2014 to June 2015. The sample size was based on a pre-study power calculation. According to reference intervals the median R value is 7.4 min (SD 1.6). According to the power calculation, the required number of participants should be 41 in order to detect a difference of 1 min with a power 80% and α of 0.05. We therefore aimed at including 41 in each group. Inclusion criteria were age ≥18 years, documented CHC infection, and compensated Child Pugh A. Fibrosis was determined using fibroscan or biopsy (cut off for no or mild fibrosis using fibroscan >8 kPa and for biopsy F1 or greater). A total of 31 patients included in the study had a Fibroscan value >12 kPa or a biopsy verified fibrosis level F4, indicating presence of cirrhosis. Exclusion criteria were anticoagulant or antithrombotic therapy, congenital hemostatic diseases including hemophilia, Hepatitis B infection, HIV infection, and autoimmune disease. Clinical characteristics for patients and controls are presented in Table 1.

The prospective study included 36 patients from the cross sectional study who initiated treatment for CHC infection. All patients were treated between eight and 24 weeks. Type and duration of treatment are shown in Table 1. At time of enrolment into the present study, 8 patients who underwent treatment against CHC, was enrolled in a clinical trial of direct acting antivirals (DAA) (MK-3682-011 and MK-3682-012). The participation in the present study did not influence participation in other studies. During treatment, two patients were diagnosed with HCC and one patient did not achieve sustained virological response (SVR). These three patients were excluded from further analyzes. Thus, the prospective cohort included 33 patients that all achieved SVR (Table 1).

The study was approved by The Committee on Biomedical Research Ethics for the Capital Region in Denmark (H-1-2014-064) and the Danish Data Protection Agency and conducted in accordance with the Second Declaration of Helsinki. Written informed consent was obtained from all participants.

### 4.2. Blood Sampling

All participants had a 30 mL venous blood sample taken at study entry. Patients included in the prospective study had an additional 30 mL venous blood sample taken at end of treatment.

### 4.3. Conventional Plasma Based and Functional Haemostatic Whole-Blood Tests

Routine biochemistry was analysed in a DS/EN ISO 15189 standardized laboratory and included D-dimer, fibrinogen (Clauss method), APTT, combined coagulation factors II-VII-X (Q hemostasis, Medinor, Denmark), antithrombin (ACL TOP, ILS, Allerod, Denmark), and platelet count (XE-2100; Sysmex, Kobe, Japan).

### 4.4. Tromboelastography (TEG)

Clot formation was assessed in 3.2% citrated whole blood (BD, Franklin Lakes, Bergen County, NJ, USA) within two hours of sampling using a TEG 5000 Haemostasis Analyzer System (Haemonetics Corp, Braintree, MA, USA) according to manufacturer’s recommendations. Variables recorded were (reference range reported by Haemonetics Corp are given in square brackets) reaction time (R (4–9 min), reflecting rate of initial fibrin formation), angle (α (55–78°), clot growth kinetics, reflecting thrombin burst), maximum amplitude (MA (51–69 mm), reflecting maximum clot strength), and lysis after 30 min (Ly30 (0–4%), proportional reduction in amplitude after MA, reflecting fibrinolysis. Day-to-day coefficient of variation of TEG MA is <7% in our laboratory. 

### 4.5. Impedance Aggregometry (Multiplate)

Whole blood platelet aggregation was analyzed in heparin anticoagulated blood by impedance aggregometry using a Multiple Platelet function Analyzer (Multiplate^®^ analyzer, Software Version 2.02.11, Dynabyte GmbH, Munich, Germany), assessing aggregation after stimulation with adenosinediphosphate test(ADPtest), arachidonic acid (ASPItest), ristocetin in high concentration (RISTOtest), and thrombin receptor agonist peptide (TRAPtest) according to manufacturer’s recommendations. Platelet aggregation was determined in response to commercially available test reagents (Dynabyte GmbH, Munich, Germany). Samples were analyzed within 30–120 min of blood sampling. All Multiplate reagents and disposables were obtained from Triolab AS (Brøndby, Denmark). Briefly, 300 μL whole blood was mixed with 300 μL NaCl (ASPItest) or NaCl-CaCl_2_ (TRAPtest, ADPtest), and 20 μL platelet agonist (TRAPtest (TRAP)-6, final concentration 32 μM), ADPtest (ADP, 6.5 μM), RISTOtest (0.77 mg/mL) and ASPItest (0.5 mM)). Increase in impedance by attachment of platelets onto Multiplate sensors is transformed to arbitrary aggregation units (AU) and plotted against time. For each applied platelet agonist, the area under the aggregation curve (AUC, AU × min or U (1 U = 10 AU × min)) after 6 min analysis was recorded by the Multiplate analyser. In order to assess platelet function “per platelet,” Multiplate result was divided by platelet count.

### 4.6. Statistics and Data

Data were analysed by SPSS Statistics 22 (IBM SPSS Statistics, Armonk, NY, USA). All data is available from authors on request. As data were not normally distributed, non-parametric tests were used. The groups included in the study (CHC with advanced or with no or mild fibrosis and uninfected controls; CHC patients before and after treatment) were compared by Mann-Whitney *U* test, Wilcoxon signed rank test, or Chi-square test/Fishers exact test as appropriate. Due to significant difference in age between groups, a Spearman correlation was performed to identify possible correlation between age and the outcomes of the study. *p*-values below 0.05 were considered to be statistical significant. 

## 5. Conclusions

In conclusion, this study found evidence for a rebalanced hemostasis in patients with CHC infection. Although having lower platelet count, impaired platelet aggregation, and lower coagulation factor II-VII-X, CHC-infected patients had normal whole blood coagulation test (TEG), possibly due to lower levels of antithombin and higher per-platelet activity, suggested by the data. Only partly normalization of platelet count, concentration of factor II-VII-X, antithrombin, and platelet activation were observed in patients achieving SVR. The implications of a rebalanced hemostasis and especially the impact on risk of CVD and bleeding warrants further studies in larger cohorts and with longer follow up.

## Figures and Tables

**Table 1:** Data are shown as medians (IQR) or *n* (%). Groups were compared with Mann-Whitney-U or chi-square test as appropriate. The indicated *p*-value represents the comparison between CHC-infected patients with no or mild fibrosis and with advanced fibrosis. IV, Intravenous; HCV, Hepatitis C virus; CHC, Chronic Hepatitis C; kPa, Kilopascal.

Baseline Characteristics	HCV Infected with No or Mild Fibrosis (*n* = 43) *	HCV Infected with Advanced Fibrosis (*n* = 39)	Healthy Controls (*n* = 39)	*p*-Value	HCV Infected + Treatment (*n* = 33)
Gender, *n* (Male) (m%)	27 (62.8)	23 (59)	20 (51.3)	0.495	21 (61.8)
Age, year, median (IQR)	49 (37–56)	57 (50–61) ^L^	51 (42–56)	0.001	55 (45–60)
Current smoker *n* (%)	18 (42) ^L^	15 (38)	10 (25)	0.358	18 (53)
HCV-RNA at inclusion, IU/mL, median, (IQR)	1.2 × 10^6^ (0.28 × 10^6^–3.00 × 10^6^)	1.6 × 10^6^ (0.68 × 10^6^–4.05 × 10^6^)	N/A	0.166	1.55 × 10^6^ (0.8 × 10^6^–3.8 × 10^6^)
Years since diagnosis of HCV infection, median (IQR)	14 (6–23)	10 (3–27)	N/A	0.266	11 (5–25)
Genotype 1/2/3/4 *n*	22/8/9/3	18/2/16/3	N/A	N/A	20/1/12/1
Fibroscan kPa, median (IQR)	5.7 (4.7–6.2)	14.1 (11.1–21.3)	N/A	<0.001	13.5 (6.6–20.2)
Bilirubin level, µmol/L, median (IQR)	7 (6–10)	11 (6–16)	N/A	0.009	11 (6–16)
Albumin level, g/L, median (IQR)	39 (37–40)	37 (34–38)	N/A	0.002	36 (34–38)
Creatinine level, µmol/L, medicn (IQR)	75 (68–86)	68 (61–80)	N/A	0.068	70 (62–82)
INR, median (IQR)	1.1 (1.1–1.2)	1.1 (1.1–1.2) ^L^	1.0 (1.0–1.1)	0.002	1.1 (1.0–1.2)
Patients with presence of portal hypertension/esophagal varices at enrollment (*n*)		5/2			4/2
Treatment used, *n* A(r+)/B(r+)/C(r+)/D(r+)/E(r+)					5(2)/17(10)/5(0)/3(0)/3(1)
Treatment duration, *n* 8/12/16/24 weeks					8/19/3/3

Treatment regimens: (**A**): sofosbuvir/simeprevir; (**B**): sofosbuvir/daclatasvir; (**C**): grazoprevir/MK-3682/MK-8408; (**D**): grazoprevir/MK-3682/elbasvir; (**E**): sofosbuvir/ledipasvir; (r+) indicating number of patients receiving the regimen including ribavirin. * 1 case without reported HCV genotype. ^L^ *p* < 0.05 by comparison with healthy controls.

**Table 2:** Data are presented as median (IQR) with p values reflecting differences between CHC-infected patients with advanced or with no or mild fibrosis. The two groups were compared Mann-Whitney *U* test. Detection limit for D-dimer was 0.3 FEU/l. D-dimer was divided into groups according to whether the individual was above or below threshold and the two groups were compared with chi-square test. The indicated p-value represents comparison between HCV infected patients with advanced and with no or mild fibrosis; arb.units, arbitrary units; CHC, Chronic Hepatitis C; FEU, Fibrinogen equivalent units; U, Units. ^L^ *p* < 0.05 by comparison with healthy controls.

Coagulation Tests	Normal Range	HCV Infected with Advanced Fibrosis	HCV Infected with no or Mild Fibrosis	*p*-Value	Healthy Controls
Standard coagulation tests					
Platelet count, median (IQR)	145–390 × 10^9^ cells/L	139 (113–187) ^L^	232 (184–267) ^L^	<0.001	254 (230–293)
Coagulation factors II-VII-X, median (IQR)	>0.60 arb.units/L	0.76 (0.6–0.86) ^L^	0.88 (0.76–1.03)	0.002	0.93 (0.81–1.06)
D-dimer, median (IQR)	>0.5 mg FEU/L	0.3 (0.3–0.4) ^L^	0.3 (0.3–0.3)	0.002	0.3 (0.3–0.3)
Above threshold, *n* (%)		8 (21) ^L^	1 (2)	0.009	0 (0)
Antithrombin, median (IQR)	0.83–1.15 × 10^3^ IU/L	0.86 (0.7–0.92) ^L^	1.01 (0.95–1.11) ^L^	0.001	1.10 (1.05–1.16)
APTT, median (IQR)	25–37 s	28 (26–30)	28 (27–30)	0.738	29 (28–31)
Fibrinogen, median (IQR)	5.3–10.3 µmol/L	8.5 (7.6–10)	8.1 (7.1–9.5) ^L^	0.333	8.7 (8.1–10.2)
Whole blood functional hemostasis tests					
R, median (IQR)	4–9 min	6,7 (5.7–7.9)	6.4 (5.5–7.5)	0.239	6.8 (5.8–7.6)
Angle, median (IQR)	55–78 degrees	65 (61–68)	67 (64–69)	0.122	67 (62–69)
MA, median (IQR)	51–69 mm	58 (54–61)	61 (57–63)	0.044	52 (56–66)
Ly30, %, median (IQR)	0–4%	0.9 (0.0–2.6)	1.4 (0.2–3.2)	0.393	1.6 (0.3–4.0)

**Table 3:** Data from 33 patients undergoing 8–24 weeks of treatment against CHC virus infection. Data are presented as medians (IQR) with p values reflecting the difference between blood results before and after treatment. The results were compared using Wilcoxon test. arb.units, arbitrary units; CHC, Chronic Hepatitis C; FEU, Fibrinogen equivalent units; U, Units. ^L^ *p* < 0.05 by comparison with healthy controls.

Coagulation in CHC-Infected Patients before and after Treatment against HCV	Pre Treatment	Post Treatment	*p*-Value	Healthy Controls
Standard coagulation tests				
Platelet count, median (IQR)	166 (113–209) ^L^	170 (118–230) ^L^	0.033	254 (230–293)
Coagulation factors II-VII-X, median (IQR)	0.79 (0.63–0.86) ^L^	0.73 (0.62–0.90) ^L^	0.837	0.93 (0.81–1.06)
D-dimer, median (IQR)	0.3 (0.3–0.4) ^L^	0.3 (0.3–0.4) ^L^	0.495	0.3 (0.3–0.3)
Above threshold (%)	5 (14) ^L^	4 (12) ^L^		0 (0)
Antithrombin, median (IQR)	0.88 (0.73–1.05) ^L^	0.93 (0.80–0.98) ^L^	0.865	1.10 (1.05–1.16)
APTT, median (IQR)	29 (27–30)	28 (26–31)	0.116	29 (28–31)
Fibrinogen, median (IQR)	8.2 (7.2–10.1)	9.6 (8.4–10.7)	0.001	8.7 (8.1–10.2)
Whole blood functional hemostasis tests				
R, median (IQR)	6.6 (5.8–7.6)	6.4 (5.5–7.2)	0.543	6.8 (5.8–7.6)
Angle, median (IQR)	65 (60–67)	66 (60–69)	0.294	67 (62–69)
MA, median (IQR)	58 (54–61)	59 (54–64)	0.058	52 (56–66)
Ly30, %, median (IQR)	0.8 (0.7–2.4)	1.2 (1.2–3.4)	0.808	1.6 (0.3–4.0)

## Supplementary Materials

Supplementary materials can be found at www.mdpi.com/1422-0067/18/5/1016/s1.

## Abbreviations

CVD	Cardiovascular disease
CHC	Chronic Hepatitis C
HCV	Hepatitis C virus
SVR	Sustained Virological Response
TEG	Tromboelastography
HCC	Hepatocellular carcinoma
vWF	Von Willebrand factor
APTT	Activated partial thromboplastin time
INR	International normalized ratio
HIV	Human immunodeficiency virus
DAA	Direct-acting antivirals
DVT	Deep vein thrombosis
EASL	European Association for the Study of the Liver
