# Supplementary material for: Impaired Platelet Aggregation and Rebalanced Hemostasis in Patients with Chronic Hepatitis C Virus Infection
Source: Int J Mol Sci. 2017 May 8;18(5):1016. doi: 10.3390/ijms18051016 (PMC5454929; doi:10.3390/ijms18051016)
Supplement: Supplementary file 1 [file ijms-18-01016-s001.pdf]

Supplementary table 1

	Normal range	HCV infected with cirrhosis (n=31)	HCV infected with no or mild fibrosis (n=43)	p-value
**Standard coagulation tests**				
Platelet count, median (IQR)	145-390 x 10^9 cells/l	139 (112-187)	232 (184-267)	<0.001
Coagulation factors II-VII-X, median (IQR)	>0.60 arb.units/l	0.77 (0.6-0.87)	0.88 (0.76-1.03)	0.006
Antithrombin, median (IQR)	0.83-1.15x10^3 IU/l	0.86 (0.7-0.92)	1.01 (0.95-1.11)	<0.001
APTT, median (IQR)	25-37 s	28 (26-30)	28 (27-30)	0.974
Fibrinogen, median (IQR)	5.3-10.3 μmol/l	8.8 (7.6-10.3)	8.1 (7.1-9.5)	0.228
**Whole blood functional hemostasis tests**				
R, median (IQR)	4-9 min	6.8 (5.8-7.9)	6.4 (5.5-7.5)	0.135
Angle, median (IQR)	55-78 degrees	65 (60-68)	67 (64-69)	0.075
MA, median (IQR)	51-69 mm	59 (54-61)	61 (57-63)	0.110
Ly30, %, median (IQR)	0-4%	0.9 (0.0-2.6)	1.4 (0.2-3.2)	0.281

Data are presented as median (IQR) with p values reflecting differences between CHC-infected patients with no or mild fibrosis and HCV patients infected with cirrhosis. The two groups were compared Mann-Whitney U test. The indicated p-value represents comparison between HCV infected patients with no or mild fibrosis and patients with cirrhosis; arb.units, arbitrary units; CHC, Chronic Hepatitis C; FEU, Fibrinogen equivalent units; U, Units
